# Proportion statistics to detect differentially expressed genes: a comparison with log-ratio statistics

**DOI:** 10.1186/1471-2105-12-228

**Published:** 2011-06-07

**Authors:** Tracy L Bergemann, Jason Wilson

**Affiliations:** 1 Division of Biostatistics, School of Public Health, University of Minnesota, Minneapolis, MN, 55455, USA; 2 Cardiac Rhythm Disease Management, Medtronic, Mounds View, MN, 55112, USA; 3 Department of Mathematics and Computer Science, Biola University, La Mirada, CA 90639, USA

## Abstract

**Background:**

In genetic transcription research, gene expression is typically reported in a test sample relative to a reference sample. Laboratory assays that measure gene expression levels, from Q-RT-PCR to microarrays to RNA-Seq experiments, will compare two samples to the same genetic sequence of interest. Standard practice is to use the log_2_-ratio as the measure of relative expression. There are drawbacks to using this measurement, including unstable ratios when the denominator is small. This paper suggests an alternative estimate based on a proportion that is just as simple to calculate, just as intuitive, with the added benefit of greater numerical stability.

**Results:**

Analysis of two groups of mice measured with 16 cDNA microarrays found similar results between the previously used methods and our proposed methods. In a study of liver and kidney samples measured with RNA-Seq, we found that proportion statistics could detect additional differentially expressed genes usually classified as missing by ratio statistics. Additionally, simulations demonstrated that one of our proposed proportion-based test statistics was robust to deviations from distributional assumptions where all other methods examined were not.

**Conclusions:**

To measure relative expression between two samples, the proportion estimates that we propose yield equivalent results to the log_2_-ratio under most circumstances and better results than the log_2_-ratio when expression values are close to zero.

## Background

Several different bioinformatics technologies exist to quantify gene expression. Regardless of technological platform, laboratory assays of gene expression first extract mRNA from a test sample and a control sample. These samples may be labeled with a tag or dye and hybridized to amplified cloned sequences that represent a gene of interest. The amount of mRNA in each sample is usually measured by examining the amount of dye remaining after hybridization. Researchers use Q-RT-PCR to measure expression when there are only one or a few genes of interest. Several lab protocols from various companies exist to quantify gene expression such as RT-PCR assays using intercalating dyes like SYBR Green, the TaqMan Gene Expression Assays, LightCycler, and QuantiGene [1-3]. When genome-wide levels of expression are of interest, microarrays can measure expression for thousands of genes of interest. Microarray platforms employ either cDNA clones [4,5] or *n*-mer oligonucleotide probes for many genes at once [6].

**Figure 1:**
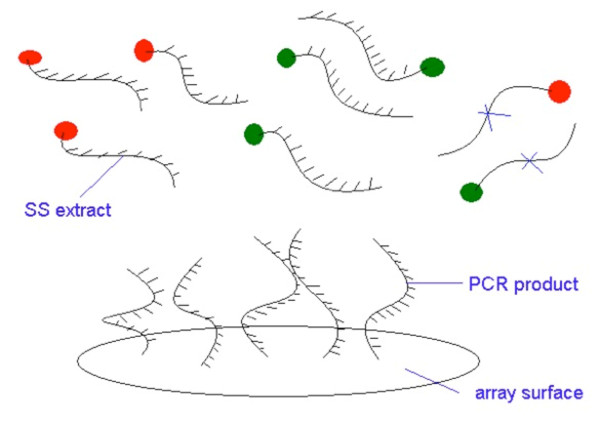
**The competitive hybridization process for a two-color system: The number of PCR products equals the number of possible hybridizations**. A proportion of the sequences will bind with matching red labeled strands and the remainder bind with the matching green labeled strands. Some sequences will not match (marked with X's) and should not hybridize.

More recently, sequence-based technologies provide more efficient and accurate expression measurements on a genome-wide scale. Evolving from early techniques such as Serial Analysis of Gene Expression (SAGE) to modern techniques such as Massively Parallel Signature Sequencing (MPSS) and RNA Sequencing (RNA-Seq), these approaches now rival microarray-based gene expression analysis for efficiency, cost, and accuracy [7]. Sequence-based techniques are also more flexible, allowing for gene expression measurements on a genome-wide level from any organism with a published genome sequence [8]. Sequencing employs systems such as the 454 or Illumina platform with the latter demonstrating greater depth and coverage [9]. To illustrate the central motive of this paper, Figure 1 demonstrates a two-color competitive hybridization assay of the kind used in TaqMan assays and cDNA microarrays. Other methods involve single-dye hybridization systems or intercalating dyes that bind to double-stranded DNA (dsDNA) product. The statistical models proposed below can be generalized to any scenario where gene expression is measured comparatively in a test sample and a reference sample.

Researchers commonly use the log_2_-ratio to measure relative mRNA expression between two samples. The estimate is as follows. Let *R_ij _*represent a summary expression value for gene *j *in the reference sample *i *where *i *= 1,..., *n *and *j *= 1,..., *K*. Let *G_ij _*represent a summary expression value for gene *j *in the test sample *i*. The value *n *is the number of paired samples or experiments and *K *is the number of genes studied. To summarize relative expression between two samples, the log_2_-ratio is(1)

or other similar variants on the theme. The log_2_-ratio is commonly interpreted as the average "log-fold-change" in gene expression between the reference sample and the test sample. Its estimate will be denoted by . If *r_j _*= 1, then the ratio between the two samples is 2^1 ^= 2, meaning that the expression of gene *j *in the test sample is two-fold that of the reference sample on average. If *r_j _*= 2, then the ratio between the two samples is 2^2 ^= 4, meaning that on average the expression in the test sample is four-fold that of the reference sample. Other values of *r_j _*are interpreted similarly.

While the interpretation of the log_2_-ratio is appealing, the statistic has an important drawback. When expression in the reference sample is low,  is numerically unstable because the denominators *R_ij _*are small. As *R_ij _*approaches zero, *r_j _*increases drastically, approaching infinity. When *R_ij _*= 0, then *r_j _*is undefined. Thus, when reference sample expression is low, we get extreme estimates or missing values for *r_j_*. This phenomenon is especially common when measuring gene expression in simple organisms. In bacteria, for example, transcription may be binary; either on or off. The log_2_-ratio is least reliable for these systems. This problem persists in human genomics research for certain experimental conditions and genes of interest.

This article proposes a new estimate to compare mRNA expression in two samples. This estimate is the proportion of mRNA in the test sample *p_j _*for each gene. The proportion takes the amount of mRNA in the test sample and compares it to the total amount of expressed mRNA represented by the sum of the test and reference samples. One formula for estimating the proportion is(2)

**Figure 2:**
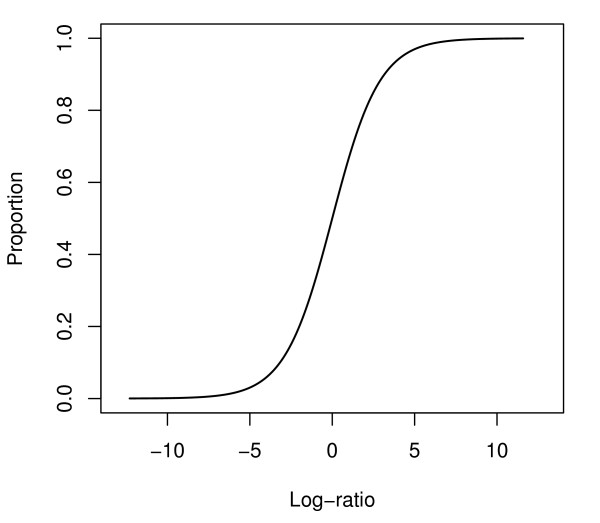
**The relationship between the log2-ratio *r_j _*and the proportion *p_j_***.

The proportion is well-defined for all values of *R_ij _*and *G_ij_*. For example, when *R_ij _*= 0, then *G_ij_/*(*G_ij _*+ *R_ij_*) = 1. We can interpret the number as follows: mRNA expression is observed only in the test sample and not in the reference sample. Similarly, if *G_ij _*= 0, then *G_ij_/*(*G_ij _*+ *R_ij_*) = 0 and this means that mRNA expression is observed in the reference sample only. Figure 2 demonstrates the relationship between the log_2_-ratio and the proportion estimates, which follows a logistic function. The relationship is roughly linear near the center point but non-linear at the extreme values. A detailed description for the estimate of the proportion, , and an alternative derived from a maximum likelihood estimate, is in the Results section.

More generally, *p_j _*can be interpreted as the proportion of mRNA from gene *j *expressed in the test sample. As *p_j _*deviates from 0.5, then there is differential expression between the test and reference samples. As *p_j _*approaches one, then gene *j *is up-regulated in the test sample. As *p_j _*approaches zero, then gene *j *is down-regulated in the test sample. The proportion statistic *p_j _*can also be transformed into a percentage: *p_j _*× 100% for reporting. For example, if *p_j _*= 0.75 then we can say that 75% of the mRNA expressed in the experiment comes from the test sample. The proportion estimate can easily be used to test for differential expression between groups. Under the null hypothesis of no gene expression, *p_j _*= 0.5. The alternative hypothesis is differential expression, *p_j _*≠ 0.5. The log_2_-ratio estimate requires a different hypothesis test. Under the null hypothesis, *r_j _*= 0 and under the alternative, *r_j _*≠ 0.

Using a proportion *p_j _*to describe relative expression for gene *j *instead of the log_2_-ratio *r_j _*maintains the ability to interpret differential expression and test for differences. The added benefit of the proportion is the ability to preserve all data points, even for experiments with very low expression values. Typically when values of *R_ij _*are very small, researchers eliminate the *j^th ^*probe of the *i^th ^*experiment from their analysis. Eliminating missing data results in a loss of information and potential bias and loss of power. The proportion estimate does not require the removal of extreme, but legitimate, data points.

The Results section provides details that describe the estimation of statistics for *p_j_*. The section also provides several test statistics for hypothesis tests of *p_j_*. Estimation and testing are developed in the frequentist context but the Bayesian context can also be used, as described in the Appendix. The Results section compares the testing scenarios in simulations and two datasets. The first dataset consists of expression data from a cDNA microarray platform and the second dataset uses RNA-Seq. Both the log_2_-ratio and proportion statistics achieve roughly equivalent results under usual conditions, but one of the proportion statistics performs better across a variety of distributional assumptions. Proportion statistics also detect differentially expressed genes that would typically be classified as missing data.

## Results

### Parameter Estimates and Hypothesis Testing

We propose a new strategy for the comparison of expression values that is tied to the underpinnings of the hybridization process and its natural interpretation using a binomial distribution. Figure 1 illustrates the hybridization process in a way that justifies the use of a binomial distribution. The description is specific to a two-color hybridization platform. The same concept extends to any system where both test samples and reference samples are assayed.

For each gene sequence, suppose that researchers amplify sequences resulting in *M_ij _*clones, where *j *is the gene probe index and *i *is the sample number, in order to co-hybridize the extracted mRNA sequences from the reference and test samples. Usually *M_ij _*is in the millions, but the exact value will be unknown. For each probe, suppose it hybridizes to a test target with probability *p_j _*and to a reference target with probability 1 - *p_j_*. This reflects the proportion of available test sequences versus references sequences. We assume that each probe must hybridize to mRNA extracted from either the test or reference sample. Then, the number of hybridizing test target sequences *Y_ij _*follows a binomial distribution with size *M_ij _*and probability *p_j_*. We wish to estimate *p_j _*to calculate the proportion of hybridized test target sequences. The maximum likelihood estimate for *p_j _*is . In this scenario, *Y_ij _*= *G_ij _*and *M_ij _*= *G_ij _*+ *R_ij _*where *R_ij _*represents the expression value for gene *j *in the reference sample *i *and *G_ij _*represents an expression value for gene *j *in the test sample *i *when there are *i *= 1,..., *n *paired experiments and *j *= 1,..., *K *genes. Therefore, to summarize *n *experiments the estimated proportion for each gene *j *is(3)

To test for differential expression we set up a decision with the null hypothesis *H*_0_: *p *= 0.5 versus the alternative hypothesis *H*_1_: *p *≠ 0.5. The test derived for this binomial distribution has a test statistic(4)

The test statistic *z_j _*is compared to a quantile from the normal distribution *z*_1-α/2_. If the type I error is *α *= 0.05, then *z*_0.975 _= 1.96. If *|z_j_| >*1.96, then gene *j *is declared differentially expressed between test and reference samples. The *z*_1-α/2 _quantile is replaced by a  quantile when the variance estimate uses  instead of *p_j_*. This test of binomial proportions, however, is not robust to deviations from the binomial distribution. Indeed, we do not believe that expression data will always follow a binomial distribution, but we include this derivation to motivate the choice of this statistic. Instead, we recommend an alternative test statistic that can be used whether distributional assumptions are met or not. The alternative test statistic simply uses a normal approximation to the binomial distribution and calculates a sample variance estimate. Then the test statistic for differential expression is(5)

where we estimate the proportion  and the sample variance in the usual way, . If |*t_j_*| >*t*_1-*α*/2,*n*-1_, then gene *j *is differentially expressed between test and reference samples. This test is valid for sufficiently large sample sizes (see Table 1).

**Table 1:** Simulation comparing test statistics for , , , limma/EBA, edgeR, and DESeq with a sample size of *n *= 20 under four distributional assumptions.

	Exponential		Poisson		Binomial		Normal	
	*fc *= 1	*fc *= 3	*fc *= 1	*fc *= 3	*fc *= 1	*fc *= 3	*fc *= 1	*fc *= 3
	0.051	0.742	0.004	0.116	0.047	1.000	0.050	1.000
+ 0.05	0.051	0.742	0.038	0.757	0.047	1.000	0.050	1.000
+ 0.5	0.051	0.742	0.044	0.943	0.047	1.000	0.050	1.000
	0.975	1.000	0.045	1.000	0.048	1.000	0.003	1.000
	0.055	0.773	0.047	0.881	0.047	1.000	0.050	1.000
	0.975	1.000	0.045	1.000	0.048	1.000	0.003	1.000
EBA	0.051	0.781	0.048	1.000	0.047	1.000	0.052	1.000
edger	NA	NA	0.033	1.000	0.014	1.000	NA	NA
DESeq	NA	NA	0.042	1.000	0.047	1.000	NA	NA

The exponential distribution has rate parameter 1/4000, the Poisson has rate parameter 3, the binomial has size 10000; and the normal has mean 10 and standard deviation 2. Each entry is proportion of times the null hypothesis was rejected at *α *= 0.05, out of 1000 simulations. The null hypothesis of no differential expression is equivalent to a fold change of one (*fc *= 1). When the fold change is three, we are calculating the power to detect differential expression (*fc *= 3). Tables for other distributional parameters may be found in Additional file 1. These tables also include a greater range of sample sizes and fold changes.

Calculating corresponding confidence intervals for each of the test statistics above is straightforward. Previous research suggests adjusting confidence intervals for binomial proportions. The most popular adjustment of these intervals uses the Agresti-Coull procedure [10,11]. We recommend this procedure to estimate confidence intervals for both of the proportion estimates above.

The proposed statistics are evaluated within a frequentist framework. A Bayesian framework is provided in the Appendix.

### Simulation Results

We ran a series of simulations to compare the inference behavior of proportion based statistics,  and , to log-ratio based statistics,  and . The proportion statistics  and  are introduced in equations 2 and 3 above and their test statistics are given in equations 4 and 5. The ratio-based statistics that have been used in the literature previously are described in equation 1 () and equation 6 in the Methods section (). In preliminary simulation exercises, we found that the performance of some test statistics was heavily dependent on the distribution used to generate the expression data. Thus, we generated expression data under four different distributions. The simulation results in Table 1 present a subset of the sample sizes and fold changes examined. More extensive tables are in Additional file 1.

The performance of the estimators under four different distributions is summarized in Table 2. The table was created after examination of the empirical type I error and power for each statistic in each simulation (Additional file 1). The proportion  performs at or above the others with the exception of  for the Poisson, although  still performs adequately there (cp. Tables 1 and 2). The statistics  and the DESeq analysis exhibit unacceptable performance under one or more of the distributional assumptions. Statistics ,  and limma/empirical Bayes (EBA) and edgeR analysis are always good or acceptable, depending on the distributional assumptions. The edgeR and DESeq analyses have type I errors less than 0.05 in many instances. On the average, the  and EBA tests have moderately better type I error and power than ,  (Additional file 1). Although  might be expected to outperform the other methods under the binomial assumption, detection under this assumption is easy and all methods performed equally well. In conclusion, the  statistic and limma/EBA have the best inference in our simulations overall. The empirical Bayes approach results in better power than  on the average under the Poisson and normal distributions.

**Table 2:** Comparison of estimators from the simulations.

	Good	Acceptable	Unacceptable
Exponential		, *EBA*	
Poisson	, *EBA*	, edgeR, DESeq	
Binomial	, EBA	edgeR	DESeq
Normal	, EBA		

Four estimators (, and ) and three methods (EBA, edgeR, and DESeq) were used under four distributional assumptions (Exponential, Poisson, Binomial, and Normal). The performance rating (Good, Acceptable, Unacceptable) was judged on the basis of Type I error and power. See Table 1 for an example of the estimators and why the ratings were judged as shown. Additional data for judging the ratings is given in Additional file 1.

### Analysis of Gene Expression in Mice with apoAI Knockout

**Figure 3:**
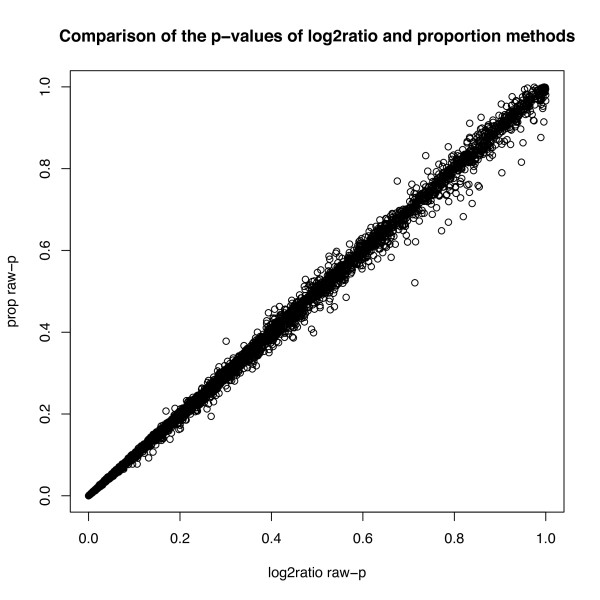
**Scatterplot of p-values for log_2_-ratio  and proportion  for the mouse data**. The general ordering of the genes is similar, although not identical, using the two methods.

To examine the performance of our method on cDNA microarray data, we analyzed the expression values reported in Ge et al (2003) [12]. Since the apoAI experiment was a control-treatment experiment that used a third sample as a reference, this data exhibits how the methods of this paper can be extended to the case of a difference of two proportions. When testing for the difference between control and treatment, the p-values from  and  were very similar in magnitude. This was true for both raw p-values and p-values adjusted for multiple-testing. The order of the p-values was also similar, but not identical (see Figure 3). When using the limma/EBA method, the p-values from  and  were again similar in magnitude, although the order varied more after the 7th probe (Table 3). The top 8 most differentially expressed probes from the original analysis differed from those selected by  using t-statistics in the 8th probe, although the top 9 probes for both sets are the same (Table 3). In the original analysis, the top 8 probes corresponded to four distinct genes, and were confirmed by real time quantitative PCR [13].

**Table 3:** Table of raw p-values for Welch t-statistics and limma methods using  and  from the apoAI control treatment expression data.

rank	p-value ()	rank	p-value ()	rank	p-value(limma ())	rank	p-value(limma ())
1	7.3 × 10^-7^	1	4.2 × 10^-6^	1	3.8 × 10^-12^	1	1.5 × 10^-9^
2	2.4 × 10^-5^	2	2.4 × 10^-5^	4	5.2 × 10^-7^	4	1.3 × 10^-6^
3	3.4 × 10^-5^	4	4.0 × 10^-5^	2	2.6 × 10^-8^	3	1.2 × 10^-7^
4	5.0 × 10^-5^	3	2.8 × 10^-5^	3	5.1 × 10^-8^	2	7.6 × 10^-8^
5	1.0 × 10^-4^	6	1.2 × 10^-4^	5	1.4 × 10^-6^	6	7.5 × 10 ^-6^

6	1.0 × 10^-4^	5	5.8 × 10^-5^	7	9.6 × 10^-6^	7	1.2 × 10^-5^
7	2.9 × 10^-4^	7	2.7 × 10^-4^	12	**4**.**5 **× **10**^-5^	11	**3**.**7 **× **10**^-5^
8	5.9 × 10^-4^	9	**6**.**4 **× **10**^-4^	8	**1**.**3 **× **10**^-5^	16	**4**.**8 **× **10**^-5^
9	7.4 × 10^-4^	8	**5**.**8 **× **10**^-4^	10	**1**.**5 **× **10**^-4^	61	**3**.**3 **× **10**^-4^
10	1.3 × 10^-3^	10	1.1 × 10^-3^	6	**2**.**0 **× **10**^-6^	5	**1**.**4 **× **10**^-6^

**rank**	**t-stat ()**	**rank**	**t-stat ()**	**rank**	**t-stat(limma ())**	**rank**	**t-stat(limma ())**

1	-16.5	1	-12.8	1	-23.1	1	-14.4
2	-9.8	2	-9.8	4	-9.0	4	-8.3
3	-9.3	4	-9.1	2	-11.5	3	-10.1
4	-8.8	3	-9.6	3	-10.9	2	-10.5
5	-7.9	6	-7.7	5	-8.2	6	-7.0
6	-7.8	5	-8.5	7	-6.7	7	-6.7

7	6.6	7	6.7	12	5.9	11	6.0
8	-5.9	9	-5.8	8	-6.7	16	-5.9
9	-5.7	8	-5.9	10	-5.2	61	-4.8
10	5.2	10	5.3	6	8.0	5	8.2

The limma method was developed for log-ratios, not proportions, but we show the results using proportions for comparison. The first group of ten rows are the p-values and the second group is the t-statistics, for reference. In the original paper, the top 8 probes were selected using the maxT multiple testing procedure with using Welch t-statistics on  in [12]. This selection is the first column, ranked 1 through 10. The p-values/t-statistics for the probes using  and limma correspond to the first column, with their ranks shown. Using t-statistics with , the selection is similar to , but not identical, since probes ranked 8th and 9th switch places. Using limma with  and , the selection begins to vary widely at the 7th probe.

When using , there were 158 (2.5%) unanalyzable probes because one or more of the samples had both *G_ij _*= 0 and *R_ij _*= 0, which made  undefined. The statistic  was defined for all probes because *G_ij _*and *R_ij _*were never zero for all samples of a specific probe. For this data, none of the 158 unanalyzed probes were in the top eight when using , although if they were a potential discovery they would have been missed using . To avoid this problem, one may add an arbitrary constant to all probes before taking the log-ratio. If merely raw p-values were selected at *α *= 0.05, then  would have selected 850, but there would have been 9 more significant p-values if an arbitrary 0.05 were added to the data to avoid zero denominators when using log-ratios. By comparison,  would have selected 871 probes.

Therefore,  is able to give comparable results to  for this cDNA microarray experiment, with the slight advantage that it provided information for 158 more probes in the study, without an arbitrary constant.

### Analysis of Differential Expression in Human Kidney and Liver Cells

To examine the performance of our methods on RNA-Seq data, we analyzed the expression values reported in Marioni et al (2008) [9]. This data compared the expression of human kidney and liver cells sampled from the same person. Concentrations of 3 pM of cDNA were sequenced using the Illumina platform in five lanes. The original paper analyzed the expression of 32,000 sequences and reported that 11,493 of the sequences were differentially expressed with q-values less than 0.001 (FDR < 0.1%) [14]. Supplemental Table 3 from Marioni et al (2008) provides the results of 17,708 sequences analyzed with both RNA-Seq technology and Affymetrix microarrays. They reported that 8,113 of Affyymetrix probe sets were differentially expressed with q-values less than 0.001.

In order to compare the methods in the original paper to those we are proposing, we used a type I error rate of *α *= 0.05/32000 for all tests. In this way, the threshold can be universally applied to all genes and methods while controlling the genomewide error rate.

Table 4 shows the total number of significant genes detected for all methods as well as the overlap between each. The least powerful method to detect differential expression was the test of  while the most powerful method was the test of . Of our proposed methods, the statistic  gave conclusions that overlapped most with the original methods in the paper, the likelihood ratio test (LRT) based on the maximum likelihood estimate [9]. In fact,  found all of the differentially expressed genes found by the LRT. Tests conducted using the edgeR package gave the second largest overlap. Comparing the proposed methods with the Affymetrix data reported in the original paper [9], the most overlap in calls was with the  tests, followed by the LRT using . If we look at the most recent methods developed for RNA-Seq data, the results of the DESeq package overlap most with , followed by the edgeR package, the LRT and then .

**Table 4:** Significant genes detected from the dataset in Marioni et al (2008).

Significant Genes in Intersecting Sets								
**Method**	**EBA (Affymetrix)**	** (LRT)**	**EBA (RNA-Seq)**	**edgeR**	**DESeq**	****		****

EBA (Affymetrix)	3641	3096	672	3127	3037	251	3183	960

(LRT)		8641	790	8461	5400	308	8641	1116

EBA (RNA-Seq)			790	790	789	275	790	700

edgeR				8697	5516	308	8697	1351

DESeq					7083	301	5644	1305

						315	308	315

							11915	3324

								3331

For each row and column, the number gives the significant gene calls by both methods. The cut-off to determine significance was set at *α *= 0.05/32000. The acronym LRT denotes the likelihood ratio test based on the Poisson distribution, as described in the Methods. The acronym EBA denotes the empirical Bayes analysis performed on both the Affymetrix and RNA-Seq data.

Log_2_-ratio estimates  produced much missing data in the RNA-Seq data analysis, with 8,947 sequences eliminated from analysis. Of these missing sequences, the proposed methods detected 4,171 () or 2,406 () significant calls (see Table 5). This means that using a test based on the log_2_-ratio may miss many possibly important differentially expressed genes. When using a LRT as suggested in Marioni et al (2008), 3,979 sequences were omitted from analysis because of missing data. The edgeR and DESeq packages did not generate any missing data. Of the 3,979 missing values generated by the LRT, our proposed methods detected 3,064 () or 2,208 () additional differentially expressed genes while the edgeR and DESeq packages detected an additional 235 and 197 genes. Since the true differential expression in this data is unknown, the differences between these methods are intriguing, but it is not clear whether one method is more accurate than another in this analysis. Overall, these findings suggest that test statistics based on a proportion statistic do not result in missing data, and more importantly, can detect possibly important differentially expressed genes that the log_2_-ratio based methods would miss.

**Table 5:** A summary of the missing values for each of the tests and the number of significant genes detected by other methods within those missing values.

Significant Genes from Sets of Missing Genes								
**Method**	**EBA (Affymetrix)**	** (LRT)**	**EBA (RNA-Seq)**	**edgeR**	**DESeq**	****	****	****

EBA (Affymetrix)	14292	561	17	589	450	16	2550	1316

(LRT)	75	3979	0	235	197	0	3064	2208

EBA (RNA-Seq)	81	0	4726	235	197	0	3064	2208

edgeR	0	0	0	0	0	0	0	0

DESeq	0	0	0	0	0	0	0	0

	422	973	5	1166	997	8947	4171	2406

	2	0	0	0	0	0	915	0

	3	0	0	0	0	0	856	1832

The diagonal gives the number of genes with missing tests. The off-diagonals indicate those genes that are significant for one method amongst the missing calls for another method. The acronym LRT denotes the likelihood ratio test based on the Poisson distribution, as described in the Methods. The acronym EBA denotes the empirical Bayes analysis performed on both the Affymetrix and RNA-Seq data.

## Discussion

Although log_2_-ratios are widely used to compare two groups of expression data, there are limitations to using these statistics. The largest drawback to ratio statistics is that they are unstable as the denominator gets closer to zero. In addition, frequentist methods for constructing a corresponding variance and formally testing hypotheses of differential expression are unsatisfying and more complicated than typical scenarios [15,16]. Due to these drawbacks, we proposed an alternative to testing for differential expression with all of the advantages of a log_2_-ratio statistic and none of its disadvantages.

We examined the proposed alternative, a proportion statistic, in four sets of simulations and two different sets of expression data. In simulations, the statistic ,  plus a constant and limma/EBA were robust to changes in distributional assumptions and the others were not. For the case of the Poisson distribution with rate parameter λ = 3, the statistic  was underpowered, but otherwise  and  performed similarly well in simulations. The simulations suggest the use of  in differential expression analyses because it uniformly preserved type I error and had competitive power. Note, however, that  is not uniformly most powerful, and statistics derived from specific distributions can beat it when the distributional assumptions hold. The performance of the empirical Bayes analysis was competitive with  in simulations but not in the analysis of the RNA-Seq dataset. Future research of interest may extend the  test within an empirical Bayes framework, akin to what already exists for the log_2_-ratio. This may even further extend the clearly demonstrated feasibility of  to detect differentially expressed genes.

Additionally, while the popular  statistic performs sufficiently well under many simulation conditions, it suffers from problems with missing data in real data analysis problems when expression values are low. The addition of constants 0.05 and 0.5 appreciably improve simulation results and make the performance of  nearly as good as  (see Additional file 1). Nevertheless, this ad hoc procedure can be avoided using . In both the analysis of a cDNA microarray set and an RNA-Seq dataset, the log_2_-ratio based statistics led to missing values. Of these genes with missing ratio values, the proportion statistic  was able to detect instances of statistically significant differential expression. We therefore recommend  for general use over the other statistics discussed.

## Conclusions

The use of the log_2_-ratio statistic to compare two expression values is challenged by denominators with near zero values. Thus, a reasonable alternative is to suggest a statistic that is not constrained by problems with very low expression values that still provides a meaningful test of differential expression. Using a proportion estimate instead of a ratio estimate does exactly that. The methods of this paper may only be used when data is naturally paired in test and reference samples, i.e. when log-ratios have traditionally been used. Our research provides several alternatives based on estimates of a proportion in both a frequentist and a Bayesian inference framework. We showed the performance of these alternatives and compared them to log_2_-ratio based tests in simulations and two gene expression datasets. In the gene expression analysis, all of the proportion methods performed better than ratio based methods for genes with low expression. For normal expression levels, inferential conclusions are similar, with the average proportion method, ,  plus a constant and the augmented log_2_-ratio method in limma/EBA, performing the best overall. The  statistic has the added advantage that it does not require adjusting for an arbitrary constant that introduces bias in the estimate. Thus, tests of differential expression should consider proportion statistics over log_2_-ratios in future scientific studies.

## Methods

This section describes the data generation process in our simulations and the data collection in the two datasets analyzed in this paper.

## Simulations

The proposed test statistics were evaluated under four different distributions. Though sophisticated simulations can be used to mimic expression data, the simulations below use simple scenarios so as to examine the performance of test statistics under basic distributions and to compare the eight different methods clearly and meaningfully. The first set of simulated intensity values were sampled from an exponential distribution that mimics the values from a 16-bit TIFF image of a cDNA microarray with respect to center and spread. The reference sample was taken from an Exp(1/4000) and the test sample was taken from a *c *× Exp(1/4000) where *c *was the fold-change value, *c *= 1,2,3,4,5. The four statistics, , and , were calculated for each value of *c *and sample sizes *n *= 3, 5, 10, 15, 20, 25, 30, 40, 50. Additionally we evaluated the ratio statistic  after shifting values for an arbitrarily small constant set at either 0.05 and 0.5. For further comparison, a standard implementation of the limma/empirical Bayes method of Smyth (2004) was perfomed [17]. For simulations of count data values, we evaluated methods that account for overdispersion in the tests of differential expression using the edgeR and DESeq packages in Bioconductor [18,19]. The implementation in both packages fixes a constant library size for each sample so that normalization is not executed. Sample sizes larger than *n *= 50 give simulation results similar to those for sample sizes of 50. In order to compare results, the p-value for an independent t-test was computed, with a null hypothesis of no difference between the two sample means. The null hypothesis was rejected if the p-value was below *α *= 0.05 and the proportion of rejections out of 1000 simulations was recorded (Table 1 and Additional file 1). The null hypothesis of no differential expression is equivalent to a fold change of one, *c *= 1. When the fold change is greater than one, we are calculating the power to detect differential expression. In this way, type I error and power were compared across the different methods. The results would be equivalent when using reciprocal fold changes instead. The simulations for an exponential distribution were repeated for an Exp(1/400) distribution, to study the effects of changing the scale.

A second set of simulated sampled intensity values from a Binomial(*M *= 10000, *p *= 0.5) distribution were obtained. The choice of this distribution was motivated by the derivation behind the maximum likelihood estimate of the proportion . The size was chosen to mimic the values from a 16-bit TIFF image of a cDNA microarray with respect to center. Analogous simulations to the exponential above were conducted with respect to statistics, sample sizes, and fold changes. For the binomial distribution, fold-changes of 2, 3, 4, and 5 correspond to binomial probabilities of 2/3, 3/4, 4/5, and 5/6 respectively. The simulations were repeated for a Binomial(*M *= 100, *p *= 0.5) distribution, to study the effects of change in the size parameter. A third set of simulated sampled intensity values from a Poisson(λ = 3) distribution were obtained. This distribution is motivated by the derivation behind the likelihood ratio test used in Marioni et al (2008) [9]. The parameter λ = 3 was chosen to mimic the number of categories arising from a smooth histogram of values from the RNA-Seq data. The simulations were repeated for a Poisson(λ = 30) distribution, to study the effects of change in the rate parameter. Analogous simulations to the exponential above were conducted with respect to statistics, sample sizes, and fold changes.

A fourth set of simulated sampled intensity values from a Normal(*μ *= 5, *σ *= 1) distribution were obtained. This distribution was included since many analyses assume expression data to be normally distributed. The center was chosen to mimic values from cDNA data with mean 5,000 and standard deviation 1,000, scaled to Normal(*μ *= 5, *σ *= 1). Analogous simulations to the exponential above were conducted with respect to statistics, sample sizes, and fold changes. For the normal distribution, fold-changes of 2, 3, 4, and 5 correspond to test samples of Normal(*c *× *μ*, *σ *= 1). The simulations were repeated for a Normal(*μ *= 10, *σ *= 2) distribution, to study the impact of changing the parameters.

All simulations were conducted using R http://www.r-project.org and the code is available in Additional file 2.

### Gene Expression Data from Mice using cDNA Microarrays

We examined our proposed approach in a well-known and often cited set of cDNA microarrays. We chose this set because many research groups have evaluated their methods on this data and consequently the differential expression behavior in this data are better understood. The Apo AI experiment used cDNA microarrays to measure gene expression in the livers of 8 inbred control mice versus 8 mice with the Apo AI gene "knocked out" [20]. For each microarray, the reference sample was created from the pooled cDNA of the eight control mice. The goal of the experiment was to detect differential expression in the liver between control mice and the genetic knockout strain [12]. Since the Apo AI gene plays a role in HDL metabolism, differentially expressed genes are likely associated with lipid metabolism. The data can be obtained as an Rdata object from http://www.bioconductor.org/help/course-materials/2005/BioC2005/labs/lab01/Data/apoai.zip on the Bioconductor website. Welch two-sample t-statistics for each of the 6,384 probes were calculated,

where *X_trt _*and *X_cont _*were either our proportion estimators,  and  or the usual log_2_-ratio estimators,  and . Since the variability of the cDNA data resembles the exponential distribution, the assumptions for methods  and  do not hold and therefore they were not used. To account for multiple testing, the original analysis used the maxT step-down procedure based on the t-statistics and found eight significantly differentially expressed probe sequences [12]. In order to explore the performance of alternative methods with both of the test statistics, the limma/EBA method of Smyth (2004) was computed [17]. Although this method was developed for log_2_-ratio values, we used the same programs on the proportion values as well.

### Gene Expression Data from Human Kidney and Liver Cells using RNA-Seq

In order to examine the performance of our new approach on a sequence-based technology, we analyzed a set of RNA-Seq data discussed in Marioni et al (2008) [9]. This set of data compared the expression of 32,000 sequences in human kidney and liver cells extracted from the same person. The expression was also measured using Affymetrix U133 oligonucleotide arrays. Data was obtained from Supplemental Table 2 in the original manuscript. To compare our methods with those reported in the Supplemental Table 3 of their manuscript, we extracted the same five lanes of Illumina sequencing data corresponding to 3 pM concentrations of cDNA. We calculated both of the proportion tests outlined in the Results section, the ratio-based test provided in the Background section, and compared them to the methods from the original paper and more recent methods that account for overdispersion [18,19].

The methods to test for differential expression from RNA-Seq data in the original paper used a likelihood ratio test (LRT) for inference [9]. Their test assumes that the expression data follows a Poisson distribution where the rate of expression λ is equivalent in kidney (K) and liver (L) cells under the null hypothesis. For gene *j*, the likelihood ratio test compares *H*_0 _: λ*_Kj _*= λ*_Lj _*versus the alternative hypothesis that expression rates differ *H*_1 _: λ*_Kj _*≠ λ*_Lj_*. The likelihood ratio test is(6)

for gene *j*. The maximum likelihood estimate for the alternative hypothesis from the above LRT is denoted by . The original paper also tested for differential expression on the Affymetrix platform for the same tissue samples. The methods employed were an empirical Bayes analysis with a false discovery rate of 0.1% [17]. More recent developments that account for overdispersion in the tests of differential expression were implemented using the edgeR and DESeq packages in Bioconductor [18,19].

## Appendix: Bayesian Estimation and Inference

In order to compare our proposed methods to previously suggested test statistics in the data analysis sections, we evaluated the proportion statistics within a frequentist testing framework. It is also possible to conceive the model in a Bayesian framework. Given the binomial assumption presented in the Results section, a Bayesian analysis can be conducted. Let the beta distribution be denoted by *β*(*a*, *b*), with density

Where Γ (*a*) is the gamma function with parameter *a*. We denote the Bayesian estimator of *p_j _*by . Using a Beta prior for *p_j _*with parameters *a *and *b*, the posterior distribution of , is *β *(*y *+ *a*, *M *- *y *+ *b*), where  and  with density

[21]. To compare the performance of the Bayesian  with frequentist statistics , and , credible intervals and confidence intervals can be constructed and coverage can be examined in simulations. For data where the difference of two proportions is required, the posterior distribution derived in [22] can be used.

## Authors' contributions

TLB and JW contributed equally to the research. Both authors read and approved the final manuscript.

## Supplementary Material

Additional file 1 **Additional materials**. Additional tables for each of the simulation scenarios are provided in the file exprPropSupp2011.pdf. This file was generated using LaTeX. Click here for file

Additional file 2 **Additional materials**. The script written in R http://www.r-project.org to conduct simulations are provided in the file exprPropSimCode.R. Click here for file
