# Supplementary material for: Proportion statistics to detect differentially expressed genes: a comparison with log-ratio statistics
Source: BMC Bioinformatics. 2011 Jun 7;12:228. doi: 10.1186/1471-2105-12-228 (PMC3224106; doi:10.1186/1471-2105-12-228)
Supplement: Additional file 1 — Additional materials. Additional tables for each of the simulation scenarios are provided in the file exprPropSupp2011.pdf. This file was generated using LaTeX. [file 1471-2105-12-228-S1.PDF]

Proportion statistics to detect differentially expressed genes: a comparison with log-ratio statistics

Supplemental materials

Tracy L Bergemann*[1,2], Jason Wilson*[3]

[1] Division of Biostatistics, School of Public Health, University of Minnesota, Minneapolis,MN, 55455, USA
[2] Cardiac Rhythm Disease Management, Medtronic, Mounds View,MN, 55112, USA
[3] Department of Mathematics and Computer Science, Biola University, La Mirada, CA 90639, USA

Email: Tracy L Bergemann*- tracy.l.bergemann@medtronic.com; Jason Wilson*- jason.wilson@biola.edu;

*Corresponding author

Supplemental Tables

Table 1

Simulation comparing test statistics for $\hat{r}, \tilde{r}, \tilde{r}+0.05, \tilde{r}+0.5, \hat{p}$, and $\tilde{p}$ as well as EBA, edgeR, and DESeq analysis under the binomial distribution assumption. The binomial distribution has size 10000. Each entry is the proportion of times the null hypothesis was rejected at $\alpha = 0.05$, out of 1000 simulations. The null hypothesis of no differential expression is equivalent to a fold change of one ($fc = 1$). When the fold change greater than one, we are calculating the power to detect differential expression.

		Fold change				
Method	N	1 (No change)	2	3	4	5
$\tilde{r}$	3	0.051	1	1	1	1
$\tilde{r}+0.05$	3	0.051	1	1	1	1
$\tilde{r}+0.5$	3	0.051	1	1	1	1
$\hat{r}$	3	0.055	1	1	1	1
$\tilde{p}$	3	0.051	1	1	1	1
$\hat{p}$	3	0.055	1	1	1	1
EBA	3	0.054	1	1	1	1
edgeR	3	0.018	1	1	1	1
DESeq	3	0.000	1	1	1	1
$\tilde{r}$	5	0.049	1	1	1	1
$\tilde{r}+0.05$	5	0.049	1	1	1	1
$\tilde{r}+0.5$	5	0.049	1	1	1	1
$\hat{r}$	5	0.051	1	1	1	1
$\tilde{p}$	5	0.049	1	1	1	1
$\hat{p}$	5	0.051	1	1	1	1
EBA	5	0.052	1	1	1	1
edgeR	5	0.018	1	1	1	1
DESeq	5	0.001	1	1	1	1
$\tilde{r}$	10	0.055	1	1	1	1
$\tilde{r}+0.05$	10	0.055	1	1	1	1
$\tilde{r}+0.5$	10	0.055	1	1	1	1
$\hat{r}$	10	0.055	1	1	1	1
$\tilde{p}$	10	0.055	1	1	1	1
$\hat{p}$	10	0.055	1	1	1	1
EBA	10	0.055	1	1	1	1
edgeR	10	0.018	1	1	1	1
DESeq	10	0.054	1	1	1	1
$\tilde{r}$	15	0.052	1	1	1	1
$\tilde{r}+0.05$	15	0.052	1	1	1	1
$\tilde{r}+0.5$	15	0.052	1	1	1	1
$\hat{r}$	15	0.048	1	1	1	1
$\tilde{p}$	15	0.052	1	1	1	1
$\hat{p}$	15	0.048	1	1	1	1
EBA	15	0.048	1	1	1	1
edgeR	15	0.020	1	1	1	1
DESeq	15	0.000	1	1	1	1

Method	N	Fold change<br>1 (No change)	2	3	4	5
$\tilde{r}$	20	0.047	1	1	1	1
$\tilde{r}+0.05$	20	0.047	1	1	1	1
$\tilde{r}+0.5$	20	0.047	1	1	1	1
$\hat{r}$	20	0.048	1	1	1	1
$\tilde{p}$	20	0.047	1	1	1	1
$\hat{p}$	20	0.048	1	1	1	1
EBA	20	0.047	1	1	1	1
edgeR	20	0.014	1	1	1	1
DESeq	20	0.047	1	1	1	1
$\tilde{r}$	25	0.050	1	1	1	1
$\tilde{r}+0.05$	25	0.050	1	1	1	1
$\tilde{r}+0.5$	25	0.050	1	1	1	1
$\hat{r}$	25	0.055	1	1	1	1
$\tilde{p}$	25	0.050	1	1	1	1
$\hat{p}$	25	0.055	1	1	1	1
EBA	25	0.054	1	1	1	1
edgeR	25	0.015	1	1	1	1
DESeq	25	0.054	1	1	1	1
$\tilde{r}$	30	0.045	1	1	1	1
$\tilde{r}+0.05$	30	0.045	1	1	1	1
$\tilde{r}+0.5$	30	0.045	1	1	1	1
$\hat{r}$	30	0.047	1	1	1	1
$\tilde{p}$	30	0.045	1	1	1	1
$\hat{p}$	30	0.047	1	1	1	1
EBA	30	0.048	1	1	1	1
edgeR	30	0.015	1	1	1	1
DESeq	30	0.046	1	1	1	1
$\tilde{r}$	40	0.055	1	1	1	1
$\tilde{r}+0.05$	40	0.055	1	1	1	1
$\tilde{r}+0.5$	40	0.055	1	1	1	1
$\hat{r}$	40	0.051	1	1	1	1
$\tilde{p}$	40	0.055	1	1	1	1
$\hat{p}$	40	0.051	1	1	1	1
EBA	40	0.052	1	1	1	1
edgeR	40	0.017	1	1	1	1
DESeq	40	0.051	1	1	1	1
$\tilde{r}$	50	0.057	1	1	1	1
$\tilde{r}+0.05$	50	0.057	1	1	1	1
$\tilde{r}+0.5$	50	0.057	1	1	1	1
$\hat{r}$	50	0.055	1	1	1	1
$\tilde{p}$	50	0.057	1	1	1	1
$\hat{p}$	50	0.055	1	1	1	1
EBA	50	0.054	1	1	1	1
edgeR	50	0.014	1	1	1	1
DESeq	50	0.053	1	1	1	1

Table 2

Simulation comparing test statistics for $\hat{r}, \tilde{r}, \tilde{r}+0.05, \tilde{r}+0.5, \hat{p}$, and $\tilde{p}$ as well as EBA, edgeR, and DESeq analysis under the binomial distribution assumption. The binomial distribution has size 100. Each entry is the proportion of times the null hypothesis was rejected at $\alpha = 0.05$, out of 1000 simulations. The null hypothesis of no differential expression is equivalent to a fold change of one ($fc = 1$). When the fold change greater than one, we are calculating the power to detect differential expression.

		Fold change				
Method	N	1 (No change)	2	3	4	5
$\tilde{r}$	3	0.048	0.806	0.971	0.992	0.996
$\tilde{r}+0.05$	3	0.048	0.806	0.971	0.992	0.996
$\tilde{r}+0.5$	3	0.048	0.808	0.972	0.992	0.997
$\hat{r}$	3	0.056	1.000	1.000	1.000	1.000
$\tilde{p}$	3	0.048	0.845	0.991	0.999	1.000
$\hat{p}$	3	0.056	1.000	1.000	1.000	1.000
EBA	3	0.037	1.000	1.000	1.000	1.000
edgeR	3	0.041	1.000	1.000	1.000	1.000
DESeq	3	0.041	1.000	1.000	1.000	1.000
$\tilde{r}$	5	0.049	0.999	1.000	1.000	1.000
$\tilde{r}+0.05$	5	0.049	0.999	1.000	1.000	1.000
$\tilde{r}+0.5$	5	0.049	0.999	1.000	1.000	1.000
$\hat{r}$	5	0.056	1.000	1.000	1.000	1.000
$\tilde{p}$	5	0.049	1.000	1.000	1.000	1.000
$\hat{p}$	5	0.056	1.000	1.000	1.000	1.000
EBA	5	0.052	1.000	1.000	1.000	1.000
edgeR	5	0.045	1.000	1.000	1.000	1.000
DESeq	5	0.045	1.000	1.000	1.000	1.000
$\tilde{r}$	10	0.046	1	1	1	1
$\tilde{r}+0.05$	10	0.046	1	1	1	1
$\tilde{r}+0.5$	10	0.046	1	1	1	1
$\hat{r}$	10	0.050	1	1	1	1
$\tilde{p}$	10	0.046	1	1	1	1
$\hat{p}$	10	0.050	1	1	1	1
EBA	10	0.046	1	1	1	1
edgeR	10	0.042	1	1	1	1
DESeq	10	0.042	1	1	1	1
$\tilde{r}$	15	0.053	1	1	1	1
$\tilde{r}+0.05$	15	0.053	1	1	1	1
$\tilde{r}+0.5$	15	0.053	1	1	1	1
$\hat{r}$	15	0.055	1	1	1	1
$\tilde{p}$	15	0.054	1	1	1	1
$\hat{p}$	15	0.055	1	1	1	1
EBA	15	0.050	1	1	1	1
edgeR	15	0.047	1	1	1	1
DESeq	15	0.047	1	1	1	1

Method	N	Fold change 1 (No change)	2	3	4	5
$\tilde{r}$	20	0.056	1	1	1	1
$\tilde{r} + 0.05$	20	0.056	1	1	1	1
$\tilde{r} + 0.5$	20	0.056	1	1	1	1
$\hat{r}$	20	0.057	1	1	1	1
$\tilde{p}$	20	0.056	1	1	1	1
$\hat{p}$	20	0.057	1	1	1	1
EBA	20	0.056	1	1	1	1
edgeR	20	0.053	1	1	1	1
DESeq	20	0.053	1	1	1	1
$\tilde{r}$	25	0.046	1	1	1	1
$\tilde{r} + 0.05$	25	0.046	1	1	1	1
$\tilde{r} + 0.5$	25	0.046	1	1	1	1
$\hat{r}$	25	0.050	1	1	1	1
$\tilde{p}$	25	0.046	1	1	1	1
$\hat{p}$	25	0.050	1	1	1	1
EBA	25	0.048	1	1	1	1
edgeR	25	0.045	1	1	1	1
DESeq	25	0.045	1	1	1	1
$\tilde{r}$	30	0.049	1	1	1	1
$\tilde{r} + 0.05$	30	0.049	1	1	1	1
$\tilde{r} + 0.5$	30	0.049	1	1	1	1
$\hat{r}$	30	0.050	1	1	1	1
$\tilde{p}$	30	0.049	1	1	1	1
$\hat{p}$	30	0.050	1	1	1	1
EBA	30	0.048	1	1	1	1
edgeR	30	0.046	1	1	1	1
DESeq	30	0.046	1	1	1	1
$\tilde{r}$	40	0.043	1	1	1	1
$\tilde{r} + 0.05$	40	0.043	1	1	1	1
$\tilde{r} + 0.5$	40	0.043	1	1	1	1
$\hat{r}$	40	0.047	1	1	1	1
$\tilde{p}$	40	0.044	1	1	1	1
$\hat{p}$	40	0.047	1	1	1	1
EBA	40	0.046	1	1	1	1
edgeR	40	0.045	1	1	1	1
DESeq	40	0.045	1	1	1	1
$\tilde{r}$	50	0.047	1	1	1	1
$\tilde{r} + 0.05$	50	0.047	1	1	1	1
$\tilde{r} + 0.5$	50	0.047	1	1	1	1
$\hat{r}$	50	0.055	1	1	1	1
$\tilde{p}$	50	0.047	1	1	1	1
$\hat{p}$	50	0.055	1	1	1	1
EBA	50	0.056	1	1	1	1
edgeR	50	0.055	1	1	1	1
DESeq	50	0.055	1	1	1	1

Table 3

Simulation comparing test statistics for $\hat{r}, \tilde{r}, \tilde{r}+0.05, \tilde{r}+0.5, \hat{p}$, and $\tilde{p}$ as well as EBA analysis under the exponential distribution assumption. The exponential distribution has rate parameter 1/4000. Each entry is the proportion of times the null hypothesis was rejected at $\alpha = 0.05$, out of 1000 simulations. The null hypothesis of no differential expression is equivalent to a fold change of one ($fc = 1$). When the fold change greater than one, we are calculating the power to detect differential expression.

Method	N	Fold change<br>1 (No change)	2	3	4	5
$\tilde{r}$	3	0.046	0.073	0.109	0.142	0.172
$\tilde{r}+0.05$	3	0.046	0.073	0.109	0.142	0.172
$\tilde{r}+0.5$	3	0.046	0.073	0.109	0.142	0.172
$\hat{r}$	3	0.974	0.985	0.991	0.994	0.997
$\tilde{p}$	3	0.082	0.134	0.204	0.272	0.334
$\hat{p}$	3	0.974	0.985	0.991	0.994	0.997
EBA	3	0.047	0.095	0.175	0.258	0.331
$\tilde{r}$	5	0.049	0.107	0.199	0.293	0.374
$\tilde{r}+0.05$	5	0.049	0.107	0.199	0.293	0.374
$\tilde{r}+0.5$	5	0.049	0.107	0.199	0.293	0.374
$\hat{r}$	5	0.973	0.987	0.996	0.998	0.999
$\tilde{p}$	5	0.071	0.148	0.272	0.379	0.463
$\hat{p}$	5	0.973	0.987	0.996	0.998	0.999
EBA	5	0.049	0.134	0.272	0.407	0.521
$\tilde{r}$	10	0.053	0.209	0.442	0.609	0.722
$\tilde{r}+0.05$	10	0.053	0.209	0.442	0.609	0.722
$\tilde{r}+0.5$	10	0.053	0.210	0.442	0.610	0.723
$\hat{r}$	10	0.972	0.993	0.998	1.000	1.000
$\tilde{p}$	10	0.060	0.230	0.467	0.639	0.744
$\hat{p}$	10	0.972	0.993	0.998	1.000	1.000
EBA	10	0.052	0.233	0.501	0.684	0.807
$\tilde{r}$	15	0.048	0.312	0.610	0.783	0.887
$\tilde{r}+0.05$	15	0.048	0.312	0.610	0.783	0.887
$\tilde{r}+0.5$	15	0.048	0.312	0.610	0.784	0.888
$\hat{r}$	15	0.972	0.996	1.000	1.000	1.000
$\tilde{p}$	15	0.052	0.326	0.632	0.811	0.905
$\hat{p}$	15	0.972	0.996	1.000	1.000	1.000
EBA	15	0.050	0.336	0.648	0.836	0.930

Method	N	Fold change<br>1 (No change)	2	3	4	5
$\tilde{r}$	20	0.051	0.397	0.742	0.893	0.958
$\tilde{r}+0.05$	20	0.051	0.397	0.742	0.893	0.958
$\tilde{r}+0.5$	20	0.051	0.397	0.742	0.893	0.958
$\hat{r}$	20	0.975	0.999	1.000	1.000	1.000
$\tilde{p}$	20	0.055	0.419	0.773	0.915	0.970
$\hat{p}$	20	0.975	0.999	1.000	1.000	1.000
EBA	20	0.051	0.414	0.781	0.926	0.974
$\tilde{r}$	25	0.052	0.463	0.832	0.952	0.984
$\tilde{r}+0.05$	25	0.052	0.463	0.832	0.952	0.984
$\tilde{r}+0.5$	25	0.052	0.463	0.833	0.952	0.984
$\hat{r}$	25	0.978	0.999	1.000	1.000	1.000
$\tilde{p}$	25	0.057	0.508	0.853	0.965	0.992
$\hat{p}$	25	0.978	0.999	1.000	1.000	1.000
EBA	25	0.052	0.488	0.856	0.965	0.992
$\tilde{r}$	30	0.050	0.548	0.897	0.975	0.994
$\tilde{r}+0.05$	30	0.050	0.548	0.897	0.975	0.994
$\tilde{r}+0.5$	30	0.050	0.548	0.898	0.975	0.995
$\hat{r}$	30	0.973	1.000	1.000	1.000	1.000
$\tilde{p}$	30	0.054	0.583	0.913	0.986	0.998
$\hat{p}$	30	0.973	1.000	1.000	1.000	1.000
EBA	30	0.048	0.564	0.912	0.984	0.998
$\tilde{r}$	40	0.049	0.662	0.958	0.995	1.000
$\tilde{r}+0.05$	40	0.049	0.662	0.958	0.995	1.000
$\tilde{r}+0.5$	40	0.049	0.662	0.959	0.995	1.000
$\hat{r}$	40	0.981	1.000	1.000	1.000	1.000
$\tilde{p}$	40	0.061	0.696	0.970	0.999	1.000
$\hat{p}$	40	0.981	1.000	1.000	1.000	1.000
EBA	40	0.048	0.674	0.969	0.997	1.000
$\tilde{r}$	50	0.044	0.768	0.984	1.000	1.000
$\tilde{r}+0.05$	50	0.044	0.769	0.984	1.000	1.000
$\tilde{r}+0.5$	50	0.043	0.771	0.986	1.000	1.000
$\hat{r}$	50	0.980	1.000	1.000	1.000	1.000
$\tilde{p}$	50	0.052	0.792	0.992	1.000	1.000
$\hat{p}$	50	0.980	1.000	1.000	1.000	1.000
EBA	50	0.042	0.787	0.989	1.000	1.000

Table 4

Simulation comparing test statistics for $\hat{r}, \tilde{r}, \tilde{r}+0.05, \tilde{r}+0.5, \hat{p}$, and $\tilde{p}$ as well as EBA analysis under the exponential distribution assumption. The exponential distribution has rate parameter 1/400. Each entry is the proportion of times the null hypothesis was rejected at $\alpha = 0.05$, out of 1000 simulations. The null hypothesis of no differential expression is equivalent to a fold change of one ($fc = 1$). When the fold change greater than one, we are calculating the power to detect differential expression.

Method	N	Fold change<br>1 (No change)	2	3	4	5
$\tilde{r}$	3	0.045	0.069	0.105	0.138	0.169
$\tilde{r}+0.05$	3	0.045	0.069	0.105	0.138	0.169
$\tilde{r}+0.5$	3	0.045	0.070	0.106	0.139	0.170
$\hat{r}$	3	0.916	0.951	0.973	0.985	0.990
$\tilde{p}$	3	0.076	0.125	0.202	0.274	0.336
$\hat{p}$	3	0.916	0.951	0.973	0.985	0.990
EBA	3	0.043	0.090	0.168	0.248	0.325
$\tilde{r}$	5	0.045	0.109	0.202	0.293	0.370
$\tilde{r}+0.05$	5	0.045	0.109	0.202	0.294	0.371
$\tilde{r}+0.5$	5	0.045	0.110	0.204	0.295	0.372
$\hat{r}$	5	0.922	0.959	0.984	0.991	0.997
$\tilde{p}$	5	0.068	0.151	0.272	0.376	0.456
$\hat{p}$	5	0.922	0.959	0.984	0.991	0.997
EBA	5	0.046	0.130	0.270	0.401	0.505
$\tilde{r}$	10	0.044	0.210	0.427	0.595	0.713
$\tilde{r}+0.05$	10	0.044	0.210	0.428	0.595	0.713
$\tilde{r}+0.5$	10	0.044	0.211	0.430	0.597	0.715
$\hat{r}$	10	0.924	0.976	0.996	0.999	1.000
$\tilde{p}$	10	0.050	0.228	0.457	0.619	0.739
$\hat{p}$	10	0.924	0.976	0.996	0.999	1.000
EBA	10	0.044	0.230	0.479	0.677	0.795
$\tilde{r}$	15	0.046	0.298	0.594	0.780	0.886
$\tilde{r}+0.05$	15	0.046	0.298	0.594	0.781	0.887
$\tilde{r}+0.5$	15	0.046	0.300	0.597	0.783	0.889
$\hat{r}$	15	0.920	0.992	0.998	1.000	1.000
$\tilde{p}$	15	0.049	0.316	0.623	0.809	0.907
$\hat{p}$	15	0.920	0.992	0.998	1.000	1.000
EBA	15	0.048	0.316	0.646	0.830	0.928

Method	N	Fold change<br>1 (No change)	2	3	4	5
$\tilde{r}$	20	0.045	0.375	0.729	0.895	0.960
$\tilde{r}+0.05$	20	0.045	0.376	0.730	0.896	0.960
$\tilde{r}+0.5$	20	0.045	0.379	0.734	0.900	0.962
$\hat{r}$	20	0.922	0.994	1.000	1.000	1.000
$\tilde{p}$	20	0.049	0.407	0.759	0.917	0.968
$\hat{p}$	20	0.922	0.994	1.000	1.000	1.000
EBA	20	0.047	0.398	0.772	0.932	0.978
$\tilde{r}$	25	0.056	0.452	0.825	0.951	0.985
$\tilde{r}+0.05$	25	0.056	0.453	0.826	0.952	0.985
$\tilde{r}+0.5$	25	0.057	0.458	0.830	0.953	0.985
$\hat{r}$	25	0.925	0.996	1.000	1.000	1.000
$\tilde{p}$	25	0.051	0.484	0.853	0.966	0.990
$\hat{p}$	25	0.925	0.996	1.000	1.000	1.000
EBA	25	0.051	0.465	0.856	0.969	0.989
$\tilde{r}$	30	0.048	0.528	0.890	0.981	0.994
$\tilde{r}+0.05$	30	0.048	0.528	0.891	0.981	0.994
$\tilde{r}+0.5$	30	0.048	0.534	0.894	0.982	0.994
$\hat{r}$	30	0.921	0.999	1.000	1.000	1.000
$\tilde{p}$	30	0.051	0.552	0.909	0.986	0.996
$\hat{p}$	30	0.921	0.999	1.000	1.000	1.000
EBA	30	0.048	0.552	0.912	0.990	0.995
$\tilde{r}$	40	0.048	0.641	0.959	0.999	1.000
$\tilde{r}+0.05$	40	0.048	0.641	0.960	0.999	1.000
$\tilde{r}+0.5$	40	0.048	0.645	0.962	0.999	1.000
$\hat{r}$	40	0.922	0.999	1.000	1.000	1.000
$\tilde{p}$	40	0.056	0.683	0.971	0.998	1.000
$\hat{p}$	40	0.922	0.999	1.000	1.000	1.000
EBA	40	0.052	0.659	0.963	0.999	1.000
$\tilde{r}$	50	0.044	0.748	0.985	1.000	1.000
$\tilde{r}+0.05$	50	0.044	0.748	0.985	1.000	1.000
$\tilde{r}+0.5$	50	0.043	0.752	0.985	1.000	1.000
$\hat{r}$	50	0.924	1.000	1.000	1.000	1.000
$\tilde{p}$	50	0.049	0.792	0.991	1.000	1.000
$\hat{p}$	50	0.924	1.000	1.000	1.000	1.000
EBA	50	0.044	0.758	0.988	1.000	1.000

Table 5

Simulation comparing test statistics for $\hat{r}, \tilde{r}, \tilde{r}+0.05, \tilde{r}+0.5, \hat{p}$, and $\tilde{p}$ as well as EBA, edgeR, and DESeq analysis under the Poisson distribution assumption. The Poisson distribution has rate parameter $\lambda = 3$. Each entry is the proportion of times the null hypothesis was rejected at $\alpha = 0.05$, out of 1000 simulations. The null hypothesis of no differential expression is equivalent to a fold change of one ($fc = 1$). When the fold change greater than one, we are calculating the power to detect differential expression.

		Fold change				
Method	N	1 (No change)	2	3	4	5
$\tilde{r}$	3	0.034	0.103	0.197	0.279	0.346
$\tilde{r}+0.05$	3	0.035	0.104	0.200	0.282	0.350
$\tilde{r}+0.5$	3	0.042	0.120	0.223	0.315	0.384
$\hat{r}$	3	0.055	0.433	0.791	0.912	0.961
$\tilde{p}$	3	0.044	0.160	0.314	0.460	0.563
$\hat{p}$	3	0.056	0.433	0.792	0.912	0.962
EBA	3	0.044	0.226	0.472	0.645	0.749
edgeR	3	0.030	0.205	0.484	0.700	0.834
DESeq	3	0.042	0.328	0.689	0.869	0.934
$\tilde{r}$	5	0.032	0.195	0.377	0.489	0.545
$\tilde{r}+0.05$	5	0.034	0.208	0.418	0.563	0.647
$\tilde{r}+0.5$	5	0.046	0.252	0.487	0.632	0.707
$\hat{r}$	5	0.055	0.596	0.914	0.982	0.995
$\tilde{p}$	5	0.048	0.291	0.538	0.676	0.737
$\hat{p}$	5	0.055	0.596	0.918	0.982	0.995
EBA	5	0.044	0.436	0.812	0.942	0.982
edgeR	5	0.030	0.376	0.765	0.911	0.969
DESeq	5	0.045	0.500	0.862	0.964	0.989
$\tilde{r}$	10	0.020	0.243	0.340	0.349	0.349
$\tilde{r}+0.05$	10	0.033	0.398	0.587	0.620	0.646
$\tilde{r}+0.5$	10	0.046	0.463	0.704	0.815	0.882
$\hat{r}$	10	0.052	0.838	0.993	1.000	1.000
$\tilde{p}$	10	0.048	0.447	0.657	0.770	0.845
$\hat{p}$	10	0.054	0.840	0.994	1.000	1.000
EBA	10	0.046	0.733	0.986	0.999	1.000
edgeR	10	0.037	0.700	0.975	0.998	1.000
DESeq	10	0.045	0.773	0.984	0.999	1.000
$\tilde{r}$	15	0.009	0.183	0.206	0.206	0.206
$\tilde{r}+0.05$	15	0.038	0.438	0.623	0.776	0.840
$\tilde{r}+0.5$	15	0.051	0.592	0.864	0.943	0.974
$\hat{r}$	15	0.048	0.942	0.999	1.000	1.000
$\tilde{p}$	15	0.050	0.554	0.814	0.892	0.923
$\hat{p}$	15	0.048	0.942	0.999	1.000	1.000
EBA	15	0.044	0.890	0.998	1.000	1.000
edgeR	15	0.037	0.870	0.998	1.000	1.000
DESeq	15	0.045	0.909	0.999	1.000	1.000

Method	N	Fold change<br>1 (No change)	2	3	4	5
$\tilde{r}$	20	0.004	0.110	0.116	0.116	0.116
$\tilde{r}+0.05$	20	0.038	0.478	0.757	0.866	0.912
$\tilde{r}+0.5$	20	0.044	0.716	0.943	0.987	0.997
$\hat{r}$	20	0.045	0.981	1.000	1.000	1.000
$\tilde{p}$	20	0.047	0.667	0.881	0.931	0.943
$\hat{p}$	20	0.045	0.981	1.000	1.000	1.000
EBA	20	0.048	0.959	1.000	1.000	1.000
edgeR	20	0.033	0.950	1.000	1.000	1.000
DESeq	20	0.042	0.968	1.000	1.000	1.000
$\tilde{r}$	25	0.003	0.068	0.068	0.068	0.068
$\tilde{r}+0.05$	25	0.040	0.549	0.832	0.926	0.962
$\tilde{r}+0.5$	25	0.048	0.801	0.980	0.998	0.999
$\hat{r}$	25	0.047	0.994	1.000	1.000	1.000
$\tilde{p}$	25	0.046	0.737	0.909	0.938	0.942
$\hat{p}$	25	0.047	0.994	1.000	1.000	1.000
EBA	25	0.047	0.985	1.000	1.000	1.000
edgeR	25	0.038	0.978	1.000	1.000	1.000
DESeq	25	0.046	0.988	1.000	1.000	1.000
$\tilde{r}$	30	0.002	0.040	0.040	0.040	0.040
$\tilde{r}+0.05$	30	0.047	0.620	0.884	0.958	0.980
$\tilde{r}+0.5$	30	0.049	0.868	0.990	0.998	1.000
$\hat{r}$	30	0.050	0.998	1.000	1.000	1.000
$\tilde{p}$	30	0.046	0.790	0.920	0.930	0.932
$\hat{p}$	30	0.050	0.998	1.000	1.000	1.000
EBA	30	0.050	0.994	1.000	1.000	1.000
edgeR	30	0.041	0.992	1.000	1.000	1.000
DESeq	30	0.046	0.996	1.000	1.000	1.000
$\tilde{r}$	40	0.001	0.014	0.014	0.014	0.014
$\tilde{r}+0.05$	40	0.047	0.724	0.955	0.990	0.996
$\tilde{r}+0.5$	40	0.050	0.945	0.999	1.000	1.000
$\hat{r}$	40	0.047	1.000	1.000	1.000	1.000
$\tilde{p}$	40	0.043	0.853	0.911	0.913	0.913
$\hat{p}$	40	0.048	1.000	1.000	1.000	1.000
EBA	40	0.044	1.000	1.000	1.000	1.000
edgeR	40	0.038	0.999	1.000	1.000	1.000
DESeq	40	0.047	1.000	1.000	1.000	1.000
$\tilde{r}$	50	0.000	0.002	0.002	0.002	0.002
$\tilde{r}+0.05$	50	0.039	0.804	0.984	0.998	1.000
$\tilde{r}+0.5$	50	0.044	0.983	1.000	1.000	1.000
$\hat{r}$	50	0.048	1.000	1.000	1.000	1.000
$\tilde{p}$	50	0.039	0.872	0.892	0.892	0.892
$\hat{p}$	50	0.049	1.000	1.000	1.000	1.000
EBA	50	0.038	1.000	1.000	1.000	1.000
edgeR	50	0.042	1.000	1.000	1.000	1.000
DESeq	50	0.050	1.000	1.000	1.000	1.000

Table 6

Simulation comparing test statistics for $\hat{r}, \tilde{r}, \tilde{r}+0.05, \tilde{r}+0.5, \hat{p}$, and $\tilde{p}$ as well as EBA, edgeR, and DESeq analysis under the Poisson distribution assumption. The Poisson distribution has rate parameter $\lambda = 30$. Each entry is the proportion of times the null hypothesis was rejected at $\alpha = 0.05$, out of 1000 simulations. The null hypothesis of no differential expression is equivalent to a fold change of one ($fc = 1$). When the fold change greater than one, we are calculating the power to detect differential expression.

		Fold change				
Method	N	1 (No change)	2	3	4	5
$\tilde{r}$	3	0.048	0.656	0.923	0.981	0.995
$\tilde{r}+0.05$	3	0.048	0.656	0.923	0.981	0.995
$\tilde{r}+0.5$	3	0.048	0.656	0.923	0.982	0.995
$\hat{r}$	3	0.048	0.998	1.000	1.000	1.000
$\tilde{p}$	3	0.049	0.699	0.960	0.996	0.999
$\hat{p}$	3	0.049	0.998	1.000	1.000	1.000
EBA	3	0.048	0.994	1.000	1.000	1.000
edgeR	3	0.040	0.994	1.000	1.000	1.000
DESeq	3	0.040	0.996	1.000	1.000	1.000
$\tilde{r}$	5	0.047	0.985	1.000	1.000	1.000
$\tilde{r}+0.05$	5	0.047	0.985	1.000	1.000	1.000
$\tilde{r}+0.5$	5	0.047	0.985	1.000	1.000	1.000
$\hat{r}$	5	0.054	1.000	1.000	1.000	1.000
$\tilde{p}$	5	0.048	0.988	1.000	1.000	1.000
$\hat{p}$	5	0.054	1.000	1.000	1.000	1.000
EBA	5	0.055	1.000	1.000	1.000	1.000
edgeR	5	0.048	1.000	1.000	1.000	1.000
DESeq	5	0.048	1.000	1.000	1.000	1.000
$\tilde{r}$	10	0.053	1	1	1	1
$\tilde{r}+0.05$	10	0.053	1	1	1	1
$\tilde{r}+0.5$	10	0.053	1	1	1	1
$\hat{r}$	10	0.052	1	1	1	1
$\tilde{p}$	10	0.054	1	1	1	1
$\hat{p}$	10	0.052	1	1	1	1
EBA	10	0.055	1	1	1	1
edgeR	10	0.048	1	1	1	1
DESeq	10	0.050	1	1	1	1
$\tilde{r}$	15	0.058	1	1	1	1
$\tilde{r}+0.05$	15	0.058	1	1	1	1
$\tilde{r}+0.5$	15	0.058	1	1	1	1
$\hat{r}$	15	0.058	1	1	1	1
$\tilde{p}$	15	0.059	1	1	1	1
$\hat{p}$	15	0.058	1	1	1	1
EBA	15	0.057	1	1	1	1
edgeR	15	0.053	1	1	1	1
DESeq	15	0.054	1	1	1	1

Method	N	Fold change 1 (No change)	2	3	4	5
$\tilde{r}$	20	0.054	1	1	1	1
$\tilde{r}+0.05$	20	0.054	1	1	1	1
$\tilde{r}+0.5$	20	0.055	1	1	1	1
$\hat{r}$	20	0.055	1	1	1	1
$\tilde{p}$	20	0.055	1	1	1	1
$\hat{p}$	20	0.055	1	1	1	1
EBA	20	0.056	1	1	1	1
edgeR	20	0.050	1	1	1	1
DESeq	20	0.053	1	1	1	1
$\tilde{r}$	25	0.053	1	1	1	1
$\tilde{r}+0.05$	25	0.053	1	1	1	1
$\tilde{r}+0.5$	25	0.052	1	1	1	1
$\hat{r}$	25	0.052	1	1	1	1
$\tilde{p}$	25	0.052	1	1	1	1
$\hat{p}$	25	0.052	1	1	1	1
EBA	25	0.058	1	1	1	1
edgeR	25	0.049	1	1	1	1
DESeq	25	0.052	1	1	1	1
$\tilde{r}$	30	0.042	1	1	1	1
$\tilde{r}+0.05$	30	0.042	1	1	1	1
$\tilde{r}+0.5$	30	0.042	1	1	1	1
$\hat{r}$	30	0.047	1	1	1	1
$\tilde{p}$	30	0.042	1	1	1	1
$\hat{p}$	30	0.047	1	1	1	1
EBA	30	0.046	1	1	1	1
edgeR	30	0.044	1	1	1	1
DESeq	30	0.046	1	1	1	1
$\tilde{r}$	40	0.044	1	1	1	1
$\tilde{r}+0.05$	40	0.044	1	1	1	1
$\tilde{r}+0.5$	40	0.044	1	1	1	1
$\hat{r}$	40	0.047	1	1	1	1
$\tilde{p}$	40	0.043	1	1	1	1
$\hat{p}$	40	0.047	1	1	1	1
EBA	40	0.049	1	1	1	1
edgeR	40	0.044	1	1	1	1
DESeq	40	0.045	1	1	1	1
$\tilde{r}$	50	0.044	1	1	1	1
$\tilde{r}+0.05$	50	0.044	1	1	1	1
$\tilde{r}+0.5$	50	0.044	1	1	1	1
$\hat{r}$	50	0.051	1	1	1	1
$\tilde{p}$	50	0.045	1	1	1	1
$\hat{p}$	50	0.051	1	1	1	1
EBA	50	0.047	1	1	1	1
edgeR	50	0.048	1	1	1	1
DESeq	50	0.049	1	1	1	1

Table 7

Simulation comparing test statistics for $\hat{r}, \tilde{r}, \tilde{r}+0.05, \tilde{r}+0.5, \hat{p}$, and $\tilde{p}$ as well as EBA analysis under the normal distribution assumption. The normal distribution has mean 5 and standard deviation 1. Each entry is the proportion of times the null hypothesis was rejected at $\alpha = 0.05$, out of 1000 simulations. The null hypothesis of no differential expression is equivalent to a fold change of one ($fc = 1$). When the fold change greater than one, we are calculating the power to detect differential expression.

		Fold change				
Method	N	1 (No change)	2	3	4	5
$\tilde{r}$	3	0.050	0.590	0.868	0.953	0.982
$\tilde{r}+0.05$	3	0.050	0.590	0.869	0.954	0.982
$\tilde{r}+0.5$	3	0.050	0.593	0.871	0.955	0.983
$\hat{r}$	3	0.000	0.714	0.999	1.000	1.000
$\tilde{p}$	3	0.051	0.636	0.924	0.984	0.996
$\hat{p}$	3	0.000	0.724	0.999	1.000	1.000
EBA	3	0.047	0.976	1.000	1.000	1.000
$\tilde{r}$	5	0.046	0.960	0.999	1.000	1.000
$\tilde{r}+0.05$	5	0.046	0.960	0.999	1.000	1.000
$\tilde{r}+0.5$	5	0.046	0.961	0.999	1.000	1.000
$\hat{r}$	5	0.000	0.961	1.000	1.000	1.000
$\tilde{p}$	5	0.048	0.965	0.999	1.000	1.000
$\hat{p}$	5	0.000	0.962	1.000	1.000	1.000
EBA	5	0.049	0.998	1.000	1.000	1.000
$\tilde{r}$	10	0.044	1	1	1	1
$\tilde{r}+0.05$	10	0.044	1	1	1	1
$\tilde{r}+0.5$	10	0.044	1	1	1	1
$\hat{r}$	10	0.000	1	1	1	1
$\tilde{p}$	10	0.044	1	1	1	1
$\hat{p}$	10	0.000	1	1	1	1
EBA	10	0.045	1	1	1	1
$\tilde{r}$	15	0.047	1	1	1	1
$\tilde{r}+0.05$	15	0.047	1	1	1	1
$\tilde{r}+0.5$	15	0.048	1	1	1	1
$\hat{r}$	15	0.000	1	1	1	1
$\tilde{p}$	15	0.047	1	1	1	1
$\hat{p}$	15	0.000	1	1	1	1
EBA	15	0.047	1	1	1	1

Method	N	Fold change 1 (No change)	2	3	4	5
$\tilde{r}$	20	0.044	1	1	1	1
$\tilde{r}+0.05$	20	0.044	1	1	1	1
$\tilde{r}+0.5$	20	0.044	1	1	1	1
$\hat{r}$	20	0.000	1	1	1	1
$\tilde{p}$	20	0.045	1	1	1	1
$\hat{p}$	20	0.000	1	1	1	1
EBA	20	0.042	1	1	1	1
$\tilde{r}$	25	0.044	1	1	1	1
$\tilde{r}+0.05$	25	0.044	1	1	1	1
$\tilde{r}+0.5$	25	0.045	1	1	1	1
$\hat{r}$	25	0.000	1	1	1	1
$\tilde{p}$	25	0.045	1	1	1	1
$\hat{p}$	25	0.000	1	1	1	1
EBA	25	0.044	1	1	1	1
$\tilde{r}$	30	0.045	1	1	1	1
$\tilde{r}+0.05$	30	0.045	1	1	1	1
$\tilde{r}+0.5$	30	0.046	1	1	1	1
$\hat{r}$	30	0.000	1	1	1	1
$\tilde{p}$	30	0.046	1	1	1	1
$\hat{p}$	30	0.000	1	1	1	1
EBA	30	0.047	1	1	1	1
$\tilde{r}$	40	0.047	1	1	1	1
$\tilde{r}+0.05$	40	0.047	1	1	1	1
$\tilde{r}+0.5$	40	0.049	1	1	1	1
$\hat{r}$	40	0.000	1	1	1	1
$\tilde{p}$	40	0.049	1	1	1	1
$\hat{p}$	40	0.000	1	1	1	1
EBA	40	0.049	1	1	1	1
$\tilde{r}$	50	0.055	1	1	1	1
$\tilde{r}+0.05$	50	0.055	1	1	1	1
$\tilde{r}+0.5$	50	0.055	1	1	1	1
$\hat{r}$	50	0.000	1	1	1	1
$\tilde{p}$	50	0.055	1	1	1	1
$\hat{p}$	50	0.000	1	1	1	1
EBA	50	0.054	1	1	1	1

Table 8

Simulation comparing test statistics for $\hat{r}, \tilde{r}, \tilde{r}+0.05, \tilde{r}+0.5, \hat{p}$, and $\tilde{p}$ as well as EBA analysis under the normal distribution assumption. The normal distribution has mean 10 and standard deviation 2. Each entry is the proportion of times the null hypothesis was rejected at $\alpha = 0.05$, out of 1000 simulations. The null hypothesis of no differential expression is equivalent to a fold change of one ($fc = 1$). When the fold change greater than one, we are calculating the power to detect differential expression.

		Fold change				
Method	N	1 (No change)	2	3	4	5
$\tilde{r}$	3	0.046	0.583	0.869	0.954	0.981
$\tilde{r}+0.05$	3	0.046	0.583	0.869	0.954	0.981
$\tilde{r}+0.5$	3	0.046	0.585	0.870	0.954	0.981
$\hat{r}$	3	0.002	0.942	1.000	1.000	1.000
$\tilde{p}$	3	0.047	0.632	0.922	0.983	0.995
$\hat{p}$	3	0.002	0.944	1.000	1.000	1.000
EBA	3	0.048	0.978	1.000	1.000	1.000
$\tilde{r}$	5	0.046	0.957	0.999	1.000	1.000
$\tilde{r}+0.05$	5	0.046	0.957	0.999	1.000	1.000
$\tilde{r}+0.5$	5	0.046	0.958	0.999	1.000	1.000
$\hat{r}$	5	0.002	0.997	1.000	1.000	1.000
$\tilde{p}$	5	0.047	0.962	0.999	1.000	1.000
$\hat{p}$	5	0.002	0.997	1.000	1.000	1.000
EBA	5	0.050	0.999	1.000	1.000	1.000
$\tilde{r}$	10	0.048	1	1	1	1
$\tilde{r}+0.05$	10	0.048	1	1	1	1
$\tilde{r}+0.5$	10	0.048	1	1	1	1
$\hat{r}$	10	0.002	1	1	1	1
$\tilde{p}$	10	0.048	1	1	1	1
$\hat{p}$	10	0.002	1	1	1	1
EBA	10	0.054	1	1	1	1
$\tilde{r}$	15	0.050	1	1	1	1
$\tilde{r}+0.05$	15	0.050	1	1	1	1
$\tilde{r}+0.5$	15	0.051	1	1	1	1
$\hat{r}$	15	0.001	1	1	1	1
$\tilde{p}$	15	0.051	1	1	1	1
$\hat{p}$	15	0.001	1	1	1	1
EBA	15	0.049	1	1	1	1

Method	N	Fold change<br>1 (No change)	2	3	4	5
$\tilde{r}$	20	0.050	1	1	1	1
$\tilde{r}+0.05$	20	0.050	1	1	1	1
$\tilde{r}+0.5$	20	0.050	1	1	1	1
$\hat{r}$	20	0.003	1	1	1	1
$\tilde{p}$	20	0.050	1	1	1	1
$\hat{p}$	20	0.003	1	1	1	1
EBA	20	0.052	1	1	1	1
$\tilde{r}$	25	0.049	1	1	1	1
$\tilde{r}+0.05$	25	0.049	1	1	1	1
$\tilde{r}+0.5$	25	0.049	1	1	1	1
$\hat{r}$	25	0.001	1	1	1	1
$\tilde{p}$	25	0.049	1	1	1	1
$\hat{p}$	25	0.001	1	1	1	1
EBA	25	0.050	1	1	1	1
$\tilde{r}$	30	0.048	1	1	1	1
$\tilde{r}+0.05$	30	0.049	1	1	1	1
$\tilde{r}+0.5$	30	0.049	1	1	1	1
$\hat{r}$	30	0.002	1	1	1	1
$\tilde{p}$	30	0.050	1	1	1	1
$\hat{p}$	30	0.002	1	1	1	1
EBA	30	0.052	1	1	1	1
$\tilde{r}$	40	0.050	1	1	1	1
$\tilde{r}+0.05$	40	0.049	1	1	1	1
$\tilde{r}+0.5$	40	0.049	1	1	1	1
$\hat{r}$	40	0.004	1	1	1	1
$\tilde{p}$	40	0.049	1	1	1	1
$\hat{p}$	40	0.004	1	1	1	1
EBA	40	0.053	1	1	1	1
$\tilde{r}$	50	0.052	1	1	1	1
$\tilde{r}+0.05$	50	0.052	1	1	1	1
$\tilde{r}+0.5$	50	0.052	1	1	1	1
$\hat{r}$	50	0.002	1	1	1	1
$\tilde{p}$	50	0.052	1	1	1	1
$\hat{p}$	50	0.002	1	1	1	1
EBA	50	0.049	1	1	1	1
